# The first reported rare case of carbamazepine-induced malignant hypertension from Nepal: a case report

**DOI:** 10.1097/MS9.0000000000002359

**Published:** 2024-07-08

**Authors:** Ashish Acharya, Kritika Bhattarai, Sarika Timilsina, Jenish Adhikari

**Affiliations:** a Nepal Medical College and Teaching Hospital, Kathmandu University; b Department of Internal Medicine, Nepal Medical College and Teaching Hospital, Kathmandu University, Kathmandu, Nepal

**Keywords:** carbamazepine, case report, malignant hypertension, Nepal

## Abstract

**Introduction and importance:**

Malignant hypertension is the most severe form of hypertension that may cause life-threatening manifestations. Carbamazepine is an antiepileptic drug primarily used for seizure disorders and trigeminal neuralgia. One of the rarely triggered adverse side effects of carbamazepine is drug-induced malignant hypertension. Here, we intend to present the first case report of carbamazepine-induced malignant hypertension from Nepal.

**Presentation of case:**

Here, we aim to present a case report of a 26-year-old female with a history of generalized tonic–clonic seizure who had developed de-novo hypertension after initiation of carbamazepine with no decrease in blood pressure to normal levels despite several antihypertensive administrations which eventually resolved on the discontinuation of drug carbamazepine. The patient was subsequently managed at our institution, where levetiracetam was used as an alternative. The patient was in close follow-up monitoring blood pressure charting.

**Clinical discussion:**

Although rare, a variety of cardiovascular side effects, including hypertension led by the drug carbamazepine, have been reported. Carbamazepine acts by inducing cytochrome P450, which facilitates an early metabolism and clearance of several antihypertensive medications, causing a decrease in their role in hypertension. The exact etiology is still debatable. However, the removal of the drug carbamazepine may result in a remission of hypertension, as illustrated in several literatures.

**Conclusion:**

Malignant hypertension is caused in rare cases to the use of the drug carbamazepine. The hypertension can undergo remission by subsequent discontinuation of the carbamazepine therapy. Regular blood pressure monitoring and charting are crucial in such cases.

## Introduction

Highlights

- Malignant hypertension is the most severe form of hypertension that may lead to life-threatening manifestations.
- Carbamazepine, an antiepileptic drug that is used for several seizure disorders treatments, has several cardiovascular side effects. Among these effects, malignant hypertension is the one which, however, occurs in the very rare minority.
- Several hypotheses exist, including the enzymatic induction of the cytochrome P450, whereas the exact pathophysiology remains debatable.
- Only a few literature are reported on carbamazepine-induced malignant hypertension. They all have reported the lowering of the patient’s elevated blood pressure to the normal level after discontinuing the drug carbamazepine, which is consistent with our case report.

Malignant hypertension is one of the most severe forms of hypertension. Hypertensive emergencies or hypertensive crisis is a potentially life‐threatening manifestation of hypertension, leading to acute impairment of more than one organ, such as large arteries, heart, kidney, and brain^1^. Originally, it was defined by two main parameters: extremely high blood pressure with diastolic blood pressure exceeding 130 mmHg at the time of the diagnosis and hypertensive retinopathy grades III or IV according to Keith *et al.*’s^2^ classification. The original definition of malignant hypertension was explicitly focused on visual disturbance but did not include evidence of other organs’ damage. These constituted various clinical presentations of the same disease entity^2^. There is very few research on malignant hypertension, and the data on its prevalence and incidence are sparse and limited. While survival after malignant hypertension has considerably improved in recent years, it still has a significant morbidity and mortality rate. Amraoui *et al.* ^3^ reported a mortality rate of 10% at 5 years in patients with an average age of 44 years. The drug-induced hypertension is found in patients taking several medications such as glucocorticoids, mineralocorticoids, sex hormones, tricyclic antidepressants, NSAIDs, and antiepileptic drugs like carbamazepine^4^. Carbamazepine is an antiepileptic drug primarily used in the management of partial seizures, generalized tonic–clonic seizures, bipolar episodes, and trigeminal neuralgia^5^.

Several adverse effects of the drug carbamazepine are found, which include drowsiness, slurred speech, ataxia, hallucination, tremors, and, in very rare cases, malignant hypertension^6^.

Here, we aim to present and discuss a case of a young woman diagnosed with malignant hypertension under carbamazepine therapy for seizure disorder and reduction of her elevated blood pressure to a normal level after discontinuing carbamazepine. This is the first rare case report of carbamazepine-induced malignant hypertension from Nepal.

We have presented the case report in accordance with 2023 SCARE Guidelines^7^.

## Case presentation

A 26-year-old young married Hindu female from the Rai ethnic group from Kathmandu, Nepal, visited the Out Patient Department of Internal Medicine of our institution in the first week of May 2023 with chief complaints of headache for a week, acute on onset, dull aching, associated with multiple episodes of nonprojectile, nonblood stained vomiting. Further, she also complained of blurring of vision that had started simultaneously. The patient gave no history of abdominal pain, decreased micturition, fever, and other constitutional symptoms. On further inquiry, the patient mentioned that she was under medication of carbamazepine 200 mg twice a day for seizure disorder, that is generalized tonic–clonic seizure for the last 2 years. She also mentioned a similar event that happened 5 months ago to her presentation that had resolved without seeking any medical attention. The patient gave no significant family history.

On examination, the patient was ill-looking, without observable signs of pallor and icterus. Bilateral pedal edema was noted up to the level of mid-leg. All her vitals were stable except blood pressure, which was found to be elevated at 260/180 mmHg and 260/140 mmHg on the left and right arms, respectively. Diagnosis of malignant hypertension was commenced, and she was concurrently admitted to the ICU of our institution. The patient was given an injection of labetalol 150 Ul/h infusion stat, with tablet amlodipine 5/50 given per oral twice a day to control her blood pressure.

Upon admission, several investigations were carried out, as listed in Table 1.

**Table 1:** The laboratory parameters of the patient.

Complete blood count: hemoglobin: 16.9 g/dl Total leukocyte count: 8860 cells/microliter Differential count: neutrophils: 77, lymphocyte: 16, eosinophils: 1 Total platelet count: 215 100 cells/microliter
Renal function test: serum urea: 24 mg/dl Serum creatinine:0.93 mg/dl Sodium: 139 mmol/l Potassium: 4.1 mmol/l e-GFR: 73 ml/min/1.73 m^2^
Urine routine microscopic examination: color: light yellow Transparency: slightly turbid Chemical reaction: acidic Albumin: 3+ Glucose: nil Pus cells: 4–5/high power field
Random blood glucose level: 117 mg/dl
Liver function test: Total bilirubin: 0.50 mg/dl Direct bilirubin: 0.09 mg/dl Indirect bilirubin: 0.41 mg/dl Alanine aminotransferase: 33 U/l Aspartate aminotransferase: 36 U/l Alkaline phosphatase: 78 U/l Total protein: 5.9 g/dl Serum albumin: 3.9 g/dl
Thyroid function test: Free tri-iodothyronine: 3.510 pg/ml, free thyroxine (FT4): 0.810 ng/dl Thyroid-stimulating hormone: 1.261 µIU/ml
Prothrombin time /INR: 15/1.07
Lipid profile: Total cholesterol: 223 mg/dl, HDL-cholesterol: 61 mg/dl LDL-cholesterol: 114 mg/dl, triglyceride: 114 mg/dl, VLDL: 48.6 mg/dl

**Figure 1:**
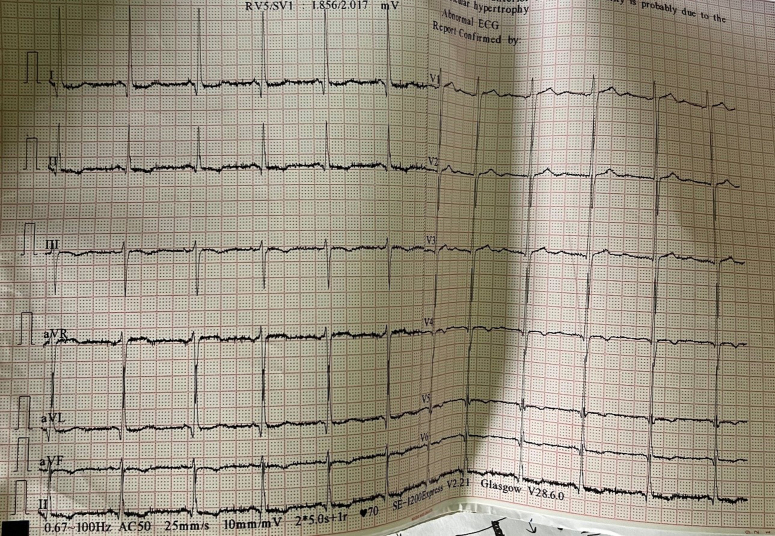
12 Lead ECG showing left ventricular hypertrophy, S wave in the lead V1, R wave in the lead V5 with >35 mm, and R wave in the lead avL with >11 mm.

ECG showed left ventricular hypertrophy on lead V1 and V5 with the lead of avL according to the Sokolow–Lyon criteria, in a sinus rhythm as shown in Figure 1.

**Figure 2:**
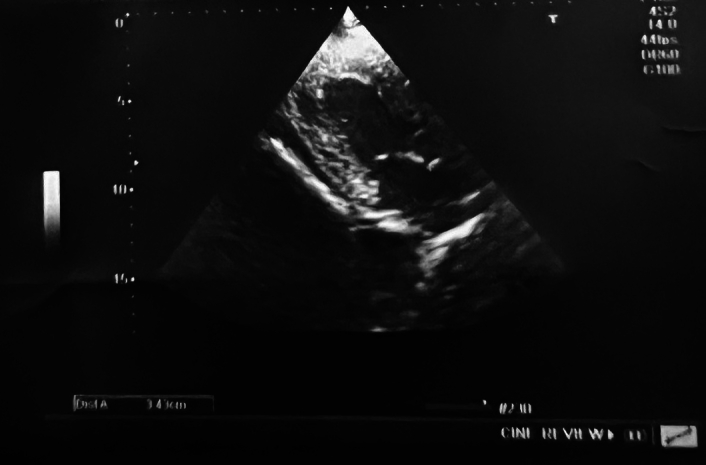
Echocardiography with a parasternal long axis view showing thickening of the left ventricular wall suggestive of left ventricular hypertrophy.

**Figure 3:**
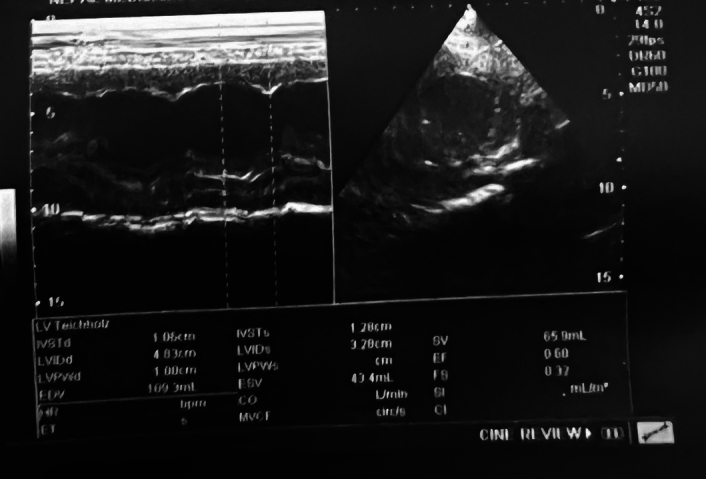
M mode measuring left ventricular end-diastolic diameter (LVEDD) and left ventricular end-systolic diameter (LVESD) of 4.83 and 3.28 cm, respectively, along with a calculated ejection fraction of 60%.

An ophthalmology consultation was accomplished for blurring of vision, the results of which revealed papilledema on both eyes, which was probably related to the extremely elevated blood pressure. Hence, an acute reduction of blood pressure, along with frequent monitoring, was advised. An echocardiography conducted by a cardiologist at our institution showed left ventricular hypertrophy, as shown in Figure 2, with left ventricular ejection fraction of 60% measured (Fig. 3).

**Figure 4:**
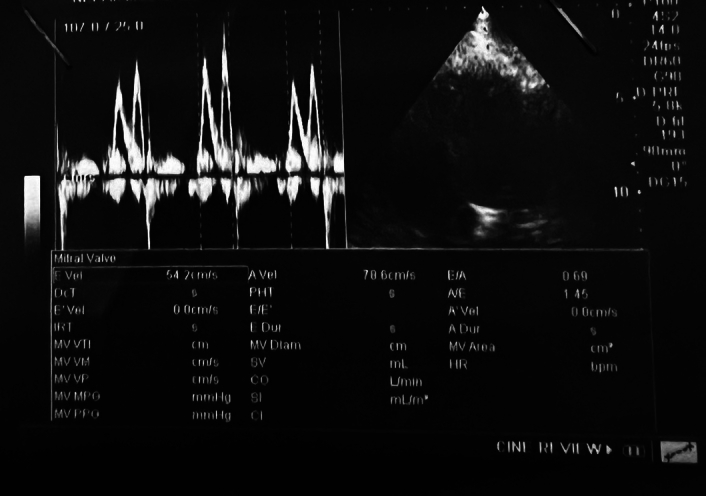
Apical chamber view in M mode measuring the E wave velocity of 54.2 cm/s and the A wave velocity of 78.6 cm/s with an E/A ratio of 0.69, labeling it as grade I diastolic dysfunction.

The E/A (indicating early and late diastolic filling velocities) ratio of 0.69 was measured, and grade I diastolic dysfunction was classified, as shown in Figure 4.

There were no valvular abnormalities with an intact interventricular and interatrial septum. The pericardium was viewed to be a normal finding.

An ultrasound sonography of the abdomen showed a right upper ureteric calculus of 10.1×4.5 cm with mild to moderate hydronephrosis. However, urosurgical consultation advised no surgical intervention until the blood pressure was fully controlled to a normal level. Similarly, renal Doppler findings indicated no evidence of renal artery stenosis. A computed tomography scan of the head showed an absence of septum pellucidum with a fusion of frontal horns of bilateral lateral ventricles and thin corpus callosum. Serology was nonreactive to hepatitis, HIV, and syphilis.

The exact cause of malignant hypertension was not properly found out after a series of tests, investigations, and analyses during her 5-day stay at the hospital. Therefore, she was discharged under antihypertensive medication.

On subsequent follow-up, her blood pressure was still at a higher side of 180/120 mmHg and 190/120 mmHg on the left and right arms, respectively. Taking into consideration the fact that the patient had had elevated blood pressure for the last 3 years but had not taken any medication for this specific disease. This hinted at the possibility of the malignant hypertension being induced due to the drug carbamazepine taken for her seizure disorder. Therefore, tablet carbamazepine was withheld, and levetiracetam 250 mg twice a day was advised as an alternative drug for her seizure disorder. Subsequently, the patient was requested for a close follow-up twice a week, and blood pressure charting was subsequently prepared and presented to us.

Her blood pressure charting in the subsequent follow-up after changing to an alternative medication of levetiracetam has been presented in Table 2.

**Table 2:** Time series blood pressure charting.

Date	Blood pressure (right)	Blood pressure (left)
03/05/2023 (at the initial time of presentation to hospital)	260/140 mmHg	260/180 mmHg
09/05/2023 (at time of discharge)	180/110 mmHg	180/110 mmHg
14/05/2023 (at 1 week follow-up)	190/120 mmHg	180/120 mmHg
21/05/2023	180/110 mmHg	170/ 110 mmHg
28/05/2023 (at the time when levetiracetam was added as an alternative)	140/100 mmHg	130/ 100 mmHg
07/06/2023	120/90 mmHg	120/90 mmHg
16/06/2023	110/80 mmHg	120/80 mmHg
25/06/2023	100/70 mmHg	100/70 mmHg
04/07/2023	110/70 mmHg	100/70 mmHg

On her last follow-up, the patient did not report any complaints of headaches or blurring of vision, and her laboratory report profile returned to normal limits. She was satisfied with the intervention and treatment that she received. The patient records her blood pressure at the home on a regular basis.

## Discussion

A young patient developed a hypertensive crisis because of the medication of drug carbamazepine for generalized tonic–clonic seizure. Although carbamazepine is an antiepileptic drug, it causes cardiovascular toxicity in overdose and to patients requiring high-maintenance doses^8^. The exact etiology of carbamazepine-induced hypertension remains unclear till date and is considered as a medical enigma. One hypothesis suggested the role of carbamazepine as being a potent enzyme inducer. Carbamazepine, along with several other several drugs, causes the enzymatic induction of cytochrome P450; this is mostly involved in the microsomal metabolism of several antihypertensive medications in the liver. Therefore, this causes a decrease in the half-life and allows the clearance of antihypertensive drugs, which has a minimal role in decreasing elevated blood pressure^9^. Carbamazepine and tricyclic antidepressants share a similar iminostilbene ring, and it has been proposed that the cardiovascular effects of carbamazepine are indistinguishable from the tricyclic antidepressants due to their molecular similarity^10^. These drugs block sodium channels, thereby decreasing the pacemaker rate and the conduction of depolarized cardiac tissues^10^. This results in variation in cardiac output. The drug has been associated with cardiovascular effects like sinus bradycardia, varying degrees of atrioventricular block, and sinus tachycardia, resulting in an increase in blood pressure^11^. Hypertension has been shown to occur with the use of tricyclic antidepressants^10^.

Bo *et al.* ^12^ suggested the possible role of antidiuretic hormone as one of the contributing factors for elevated blood pressure due to the use of carbamazepine. Their case study was similar to ours, where elevated blood pressure was only found when carbamazepine was taken and its subsequent decrease after discontinuation of the drug.

Only a few studies have been published regarding carbamazepine-induced hypertension^9,10,12–17^, and their results are consistent with ours, where the diagnosis was given after the exclusion of other likely possible causes of malignant hypertension.

This case is the first case of carbamazepine-induced malignant hypertension to be reported from Nepal. Our patient was classified with grade I diastolic dysfunction after the calculation of the E/A ratio, which was measured to be 0.69. The normal E/A ratio value ranges from 0.75 to 1.5, although this depends upon the age and sex of the patient. A lower value of the E/A ratio indicates a diastolic dysfunction, which may probably result in due to elevated blood pressure^18^.

After the withdrawal and subsequent replacement of carbamazepine by levetiracetam, the patient had no signs of elevated blood pressure or hypertension in subsequent follow-ups at our institution, which typically suggests a clear relation between the drug carbamazepine and hypertension.

### Take away lessons

One of the vital knowledge that we can gain from the current case report is that drug-induced malignant hypertension (carbamazepine) is rare but can lead to significant morbidity and mortality. Although cases of malignant hypertension are rare and insignificant in regular hospital settings, they should be ruled out, especially in those who are under carbamazepine for seizure disorder having a history of hypertension.

### Strength and limitation

This is the first reported case among the Nepalese population despite challenges encountered in diagnosing the disease due to limited available resources. A further investigation and analysis of why and how carbamazepine causes malignant hypertension could not be accomplished due to the lack of respective facilities at our institution. Nevertheless, we hope that the case report will add vital information to the existing literature on carbamazepine-induced malignant hypertension.

## Conclusion

Carbamazepine is one of the antiepileptic drugs used for seizure disorder, trigeminal neuralgia, and bipolar disorder. Several adverse reactions do occur due to its use, however, cardiovascular side effects are primarily seen because of overdoses and long-term maintenance therapies. Malignant hypertension, which is also termed as hypertensive crisis, occurs in the rare due to the use of carbamazepine. Several hypotheses on malignant hypertension exist, but the exact reason behind its occurrence still remains unclear. Malignant hypertension can undergo remission because of subsequent discontinuation of carbamazepine therapy, as documented in our case. However, regular blood pressure monitoring and charting are crucial in such cases of hypertension.

## Ethical approval

Case reports are exempt from ethical approval in our institution, Nepal Medical College and Teaching Hospital, Attarkhel.

## Consent

Written informed consent was obtained from the patient for publication of this case report and accompanying images. A copy of the written consent form is available for review by the editor-in-chief of this journal upon request.

## Source of funding

There are no sources of funding.

## Author contribution

All the authors contributed equally to the preparation of this case report. A.A., K.B., S.T.., and J.A. were involved in conceptualization, study design, previous literature review, collection of all the information from the patient with echocardiographic and ECG images, and laboratory reports, requisition of written consent, preparation of manuscript, and editing. All the authors individually did a final proofreading of the manuscript before submission.

## Conflicts of interest disclosure

The authors declare no conflicts of interest.

## Research registration unique identifying number (UIN)

Not applicable.

## Guarantor

Ashish Acharya.

## Data availability statement

Not applicable.

## Provenance and peer review

Not commissioned, externally peer-reviewed

## Disclosure

This case has not been presented in a conference or regional meeting and is not under consideration at any other journal.
